# The Potential of *Trichoderma*-Mediated Nanotechnology Application in Sustainable Development Scopes

**DOI:** 10.3390/nano13172475

**Published:** 2023-09-01

**Authors:** Ali Athafah Tomah, Zhen Zhang, Iman Sabah Abd Alamer, Arif Ali Khattak, Temoor Ahmed, Minjun Hu, Daoze Wang, Lihui Xu, Bin Li, Yanli Wang

**Affiliations:** 1 State Key Laboratory for Quality and Safety of Agro-Products, Institute of Plant Protection and Microbiology, Zhejiang Academy of Agricultural Sciences, Hangzhou 310021, China; ali_athafah@uomisan.edu.iq (A.A.T.); zhangz@zaas.ac.cn (Z.Z.); 2 State Key Laboratory of Rice Biology and Breeding, Ministry of Agriculture, Key Laboratory of Molecular Biology of Crop Pathogens and Insects, Institute of Biotechnology, Zhejiang University, Hangzhou 310058, China; 11716107@zju.edu.cn (I.S.A.A.); 11618404@zju.edu.cn (A.A.K.); temoorahmed@zju.edu.cn (T.A.); libin0571@zju.edu.cn (B.L.); 3 Plant Protection, College of Agriculture, University of Misan, Al-Amarah 62001, Iraq; 4 Plant Protection, Agriculture Directorate, Al-Amarah 62001, Iraq; 5 Xianghu Laboratory, Hangzhou 311231, China; 6 Agricultural Technology Extension Center of Fuyang District, Hangzhou 311400, China; minjunhu48@gmail.com; 7 Hangzhou Rural Revitalization Service Center, Hangzhou 310020, China; wdz2005@163.com; 8 Institute of Eco-Environmental Protection, Shanghai Academy of Agricultural Sciences, Shanghai 201403, China

**Keywords:** *Trichoderma* spp., cell-free culture filtrate, mycosynthesis, nanoparticles, eco-friendly, low cost, safer

## Abstract

The environmental impact of industrial development has been well-documented. The use of physical and chemical methods in industrial development has negative consequences for the environment, raising concerns about the sustainability of this approach. There is a growing need for advanced technologies that are compatible with preserving the environment. The use of fungi products for nanoparticle (NP) synthesis is a promising approach that has the potential to meet this need. The genus *Trichoderma* is a non-pathogenic filamentous fungus with a high degree of genetic diversity. Different strains of this genus have a variety of important environmental, agricultural, and industrial applications. Species of *Trichoderma* can be used to synthesize metallic NPs using a biological method that is environmentally friendly, low cost, energy saving, and non-toxic. In this review, we provide an overview of the role of *Trichoderma* metabolism in the synthesis of metallic NPs. We discuss the different metabolic pathways involved in NP synthesis, as well as the role of metabolic metabolites in stabilizing NPs and promoting their synergistic effects. In addition, the future perspective of NPs synthesized by extracts of *Trichoderma* is discussed, as well as their potential applications in biomedicine, agriculture, and environmental health.

## 1. Introduction (General Properties of *Trichoderma*)

Although the genus *Trichoderma* was first discovered by Persoon in 1794, it was not until Weindling published his first full paper on *Trichoderma lignorum* in 1934 that its role as a biological control agent was realized. Weindling’s paper showed that this species can control plant diseases, and this discovery led to a renewed interest in *Trichoderma* as a potential biocontrol agent [1]. *Trichoderma* is the asexual stage of the filamentous Hypocrea genus belonging to the Ascomycota fungi division. The species of this genus are free-living saprophytic fungi found in all soils, with an average presence in temperate and tropical soils of nearly 101-103 culturable propagules per gram [2,3]. These fungi reproduce asexually by the production of conidia and chlamydospores and in wild habitats by ascospores [4]. The genus *Trichoderma* is one of the most frequently isolated soil microorganisms. It has several useful properties, including being non-pathogenic to humans and plants, environmentally friendly, easy to isolate and culture, capable of rapid growth on a variety of inexpensive organic substrates, and able to produce a wide range of proteins, enzymes, and secondary metabolites [5,6,7]. The species of this genus are genetically quite diverse, with differences in capabilities among strains. These fungi have been widely used as biocontrol agents. Some *Trichoderma* spp. are known to control plant diseases through a variety of mechanisms, either indirectly (by competing for nutrients and space, modifying the environmental conditions, or promoting plant growth and plant defense mechanisms and antibiosis) or directly (by mechanisms such as mycoparasitism or the synergistic action of several mechanisms) [8]. *Trichoderma* spp. produce a variety of antibiotics and secondary metabolites that play an important role in inhibiting pathogens. The antibiotic’s mechanism is via low-molecular-weight diffusible organic compounds excreted by *Trichoderma* that inhibit the growth of the pathogen. The bioactive compounds secreted by *Trichoderma* are natural compounds that are chemically different, non-polar, and low molecular mass (less than 3000 Daltons) [9,10] and include polyketides, alkaloids, terpenoids, non-ribosomally biosynthesized peptides (NRPs), and metabolites of mixed biogenesis [10,11]. The compounds, which are typically composed of 5–20 amino acid residues, have a high content of α-aminoisobutyric acid (Aib) with an acylated N-terminus and a complex C-terminus that may consist of a free or methoxy-substituted 2-amino alcohol, amine, amide, free amino acid or sugar alcohol [12,13].

The antibiotic production of over 180 secondary metabolites, representing different classes of chemical compounds exhibiting biocontrol activity, has been reported for isolates of *Trichoderma* [10,14]. For example, gliovirin is produced from *Gliocladium virens* and has antimicrobial active against of *Pythium ultimum* [15], while gliotoxin from *T. virens* [16] inhibits the mycelium of both *P. ultimum* and *Rhizoctonia solani* [17]. Some species produce types of peptaibols (linear peptides), such as trichokonin VI from *T. pseudokoningii* SMF2, which has a wide antimicrobial spectrum against several bacteria, yeasts, and filamentous fungi [18], and trichorzianine from *T. harzianum*, which exhibits antibacterial activity against *S. aureus* [19], while koninginin A and B (polyketides group) are secreted by *T. koningii* [20,21]. Pyrone 6-PP was first discovered in a culture broth of *T. viride* [22] and is classified as a volatile organic compound. The 6-PP compound displayed good antifungal activity against *Aspergillus flavus*, *Penicillium expansum*, and *Fusarium acuminatum* [23]. Other antifungal compounds isolated from *Trichoderma* spp., belonging to different chemical classes, have been used in plant protection as an environmentally friendly and efficient management tool against a variety of phytopathogens.

These metabolites can be either overproduced or combined with other metals to create new formulations that are more efficient for use in several fields, including in the control of plant diseases. Biomolecules can bind to metals through their proteins and amino acid residues, forming a coating on the surface of the NPs. This coating, known as capping, can increase the stability of the NPs and prevent them from aggregating [24,25]. Subsequent stabilization can also be provided by free amino groups or cysteine residues or through the electrostatic attraction resulting from negative carboxyl groups provided by mycelial cell wall enzymes present in the filtrate. Furthermore, the ability of the thiol (-SH) group to form disulfides—perhaps the most important bridging structure in nature—with cysteine subunits of endogenous proteins renders them unique among the functional groups utilized in nanotechnology to form thiolated disulfide bonds that tightly bind with noble metals, leading to the formation of stable NPs [24,26,27]. For example, FTIR analysis confirmed that a gliotoxin bioactive compound secreted by *T. virens* is responsible for capping and reducing Ag^+^ and the biosynthesis of silver NPs through binding of each of the negative carboxyl groups, sulfur, and oxygen from the hydroxyl groups of gliotoxin with silver [28].

The main objective of this review is to highlight the *Trichoderma* fungus as a rich source of secondary metabolites and their role in the synthesis of eco-friendly NPs. The review also highlights the emerging combination of noble metals and *Trichoderma* derivatives, the immense potential produced by synergistic interactions between them, and the extent of their application in the fields of agriculture, industry, the environment, and health safety.

## 2. Trichogenic Nanoparticles

The term “mycosynthesis” was first used to describe the synthesis of NPs using the fungus *F. acuminatum* by Ingle et al. [29]. Since then, the term “Myconanotechnology” has expanded to include literature studies that describe NPs synthesized using fungi [30]. The term “Trichogenic” has recently been used to describe the biogenic synthesis of selenium NPs using six species of *Trichoderma* [31].

Among the most important fungi that are used in biological control against phytopathogens, the *Trichoderma* species have the potential to be used to synthesize NPs on a large scale by using an environmentally friendly production process. According to the inventory list conducted by Cai and Druzhinina [32], there are around 460 species with valid names in the *Trichoderma* genus that have been deposited in public updated databases available on the International Subcommission on Taxonomy of *Trichoderma* website (www.trichoderma.info, accessed on 25 February 2022). However, and from a thorough inventory of literature studies, we found that only about 13 species belonging to the *Trichoderma* genus show the ability for NP synthesis, including *T. harzianum*, *T. asperellum*, *T. viride*, *T. atroviride*, *T. virens*, *T. longibrachiatum*, *T. pseudokoningii*, *T. reesei*, *T. koningii*, *T. brevicompactum*, *T. citrinoviride*, *T. hamatum*, and *T. gamsii*. These species are known to be applied globally as biological control agents against different plant pathogens. Although the number of *Trichoderma* species has been increasing, research on the synthesis of NPs using this genus is still in its early stages. More research is needed to explore the potential of *Trichoderma* species for nanoparticle synthesis.

**Figure 1:**
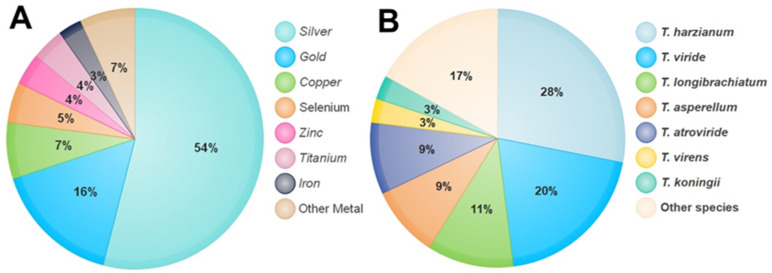
The percentage of metals used in the synthesis of NPs by *Trichoderma* species (**A**). Percentage of *Trichoderma* species used in the synthesis of NPs (**B**).

The first report on the use of *Trichoderma* in the synthesis of silver NPs by Mukherjee [33] dates back to 2008. This strategy has attracted much more attention in the most recent decade as the synthesis mechanism for various NPs using diverse metals such as silver (Ag), gold (Au), copper (Cu), zinc (Zn), and so on. NPs of both silver and gold are considered more secure in contrast to other metallic NPs [34]. Through a survey of the literature published from the years 2008 to 2021, more than 100 research papers published in the approved journals adopted the use of *Trichoderma* in the synthesis of NPs from different metals. Using the percentage equation [(M/N) × 100)], where M means the number of studies that used *Trichoderma* in the synthesis of NPs from a specific metal and N means the number of research studies that used *Trichoderma* in the synthesis of NPs from different minerals, the proportional analysis shows that silver metal occupied the leading position in the synthesis of NPs by the *Trichoderma* species, which was 54% of the total of other metals (Figure 1A). On the other hand, most of the *Trichoderma* spp. applied in biological control were suggested to have the capacity to biosynthesize NPs. Five different species of *Trichoderma*, viz., *T. asperellum*, *T. harzianum*, *T. longibrachiatum*, *T. pseudokoningii*, and *T. virens*, led in the production of silver NPs (AgNPs) [35]. Spherical AgNPs with sizes from 2 to 15 nm were formed using cell filtrates of *T. inhamatum* [36]. Production of silver NPs was achieved through extracellular reduction by six isolates of *T. virens*, and a single and aggregated form was obtained, which was uniform in shape and had a size of 8–60 nm [37]. Meanwhile, six species belonging to the *Trichoderma* genus, viz., *T. asperellum*, *T. harzianum*, *T. atroviride*, *T. virens*, *T. longibrachiatum*, and *T. brevicompactum*, were successful in the biogenic synthesis of selenium NPs (SeNPs) [31].

Also, using the extracellular filtrate of *T. viride* to yield of AuNPs, a mixture of spheres, triangles, hexagons, and rod shapes with sizes 20–30 nm were obtained, with a few as big as 120 nm [38]. Meanwhile, the AuNPs obtained using the *T. asperellum* biomass were well dispersed, with diverse shapes such as spheres, triangles, and hexagons [39].

A *T. hamatum* cell-free filtrate is used for the synthesis of AuNPs of dimensions 5–30 nm and with diverse shapes such as spheres, pentagons, and hexagons [40]. Additionally, magnesium oxide (MgO) NPs at a size ranging from 45.12 to 95.37 nm were obtained using the extracellular approach of *T. viride* [41], zinc oxide (ZnO) NPs with a mean size of 30.34 nm were biosynthesized using the fungal mycelial water extract derived from *T. harzianum* [42], copper oxide (CuO) NPs with crystalline and spherical-shaped particles with an average size of 110 nm were produced using a cell-free extract of *T. asperellum* [43], and the biosynthesis of cadmium sulfide (CdS) NPs with a size range of 3–8 nm and a spherical morphology has been already reported using the fungal biomass of *T. harzianum* [44]. Titanium oxide (TiO_2_) NPs of highly irregular size (10–400 nm) and triangular, pentagonal, spherical, and rod-like shapes were synthesized using TiO_2_ with the extract of *T. citrinoviride* as a reducing agent [45]. A literature survey of research papers published from 2008 to 2021 in approved journals revealed that more than 100 studies had adopted the use of *Trichoderma* in the synthesis of NPs. Using the percentage equation [(T/N) × 100)], where T means the number of studies that used one species of *Trichoderma* in the synthesis of NPs, while N means the number of research that used all different types of *Trichoderma* in the synthesis of NPs, the proportional analysis shows the potential of most *Trichoderma* species in the biosynthesis of NPs by the reduction of a wide range of diverse metals (Figure 1B).

Among the interesting metals, selenium (Se), and its applications in biomedicine, agriculture, and environmental health, has become of great research interest in recent decades. *Trichoderma* has been used in the formation of nanoproducts by selenium metal due to its ability to reduce selenite and convert it into less toxic derivatives [46].

Selenium (Se) NPs are gaining importance in the field of medicine owing to their antibacterial and anticancer properties [47]. SeNPs at a size of 20–220 nm and spherical and pseudo-spherical Se particles were synthesized by the SeO_2_ reduction process in the *Trichoderma* spp. WL-Go culture broth and the pure product were obtained and characterized through filtration of the culture broth supernatant [48]. SeNPs, as plant biostimulants, were obtained using the filtrate of *Trichoderma* strains to mediate the reduction of selenium (Se) ions, and their effects on different stages of *Vigna radiata* plant growth were observed. Besides the growth improvement and protection of the plants from phytopathogens, SeNPs were found to be much less toxic than Se selenite in the tested seeds [49]. Inoculated plants with biosynthesized SeNPs from *T. atroviride* exhibited a significant level of protection of 72.9% against late blight of tomato caused by *P. infestans*, where the defense responses noted included an accumulation of lignin, callose, and hydrogen peroxide that supported the cellular defense mechanism, while the biochemical defense mechanism collaborated by elevating the levels of lipoxygenase (LOX), phenylalanine lyase (PAL), β-1,3-glucanase (GLU), superoxide dismutase (SOD) in plants [50]. The selenium (Se) NPs synthesized using the *Trichoderma* sp. fungus culture filtrate showed great effectiveness as a larvicidal and antifeedant agent at a concentration of 100 µg/mL for 48 h, and they can be used for the control of *Spodoptera litura* larvae that infect peanut and castor plants [51]. Acknowledging these Se advantages, the synthesis of SeNPs via *Trichoderma* is preferred as it offers a safer, more eco-friendly, and less toxic alternative compared with other metals. Additionally, biologically synthesized SeNPs tend to be more stable as they have a natural coating of organic material on their surface, which will prevent the aggregation of the NPs over a long period of time [51].

## 3. Contribution of Active *Trichoderma* Derivatives to NPs Formation

Many important features of *Trichoderma* species have allowed them to excel in a variety of scientific fields, such as agriculture, medicine, and industrial processes [52,53,54]. Biomolecules derived from *Trichoderma* organisms play an important role in the synthesis of nanoparticles. They act as capping agents, which help to stabilize the NPs and make them less toxic. This is because the biomolecules prevent the NPs from aggregating and releasing harmful substances [55,56], allowing them to present greater biological activity [57]. Recently, organic molecules such as proteins, amino acids, organic compounds, and functional groups from proteins, such as -OH, -COOH, -SH, -NH_2_, -PO_4_, etc., that are secreted by these species were found to play a role in NP synthesis by providing binding sites for the metal ions followed by their reduction into NPs [31,48,58]. Another important discovery was that amino protein groups are not exposed to secondary and tertiary structure distortions or rupture of their covalent bonds during the process of reduction and binding to the surface of silver NPs [59], which indicates that these bands of proteins retain their biological functions, thus participating in the biological activity with NPs. Based on that, characteristics of the levels of toxicity to NPs are differentiated depending on the reducing agents and biological stabilizers that result in different coatings [60]. In this context, the advantage of using the proteins and metabolites of the fungal *Trichoderma* spp. that are involved in the biological control of phytopathogens in NP capping is more convincing and safer for the environment [61]. Also, the biological synthesis of NPs using proteins and metabolites of the fungus *Trichoderma* as a biological control agent of phytopathogens contributes to the synergistic activity between the metal formed and the capping agent present on it as an inhibitor of microbes [62]. Silver NPs from metabolites of the cell-free filtrate of *T. viride* (MTCC 5661) were synthesized.

FTIR analysis demonstrated the role of extracellular secondary metabolites and proteins of *T. viride* in the capping of Ag NPs, and the type of secondary-metabolite-coated silver NP was identified by GC-MS, which found that 16 antimicrobial metabolites comprised of organic acids, sugars, intermediates of carbohydrate metabolism, and amino acids and their derivatives were involved in the coating of silver NPs. In comparison with citrate-stabilized silver NPs, biologically synthesized silver NPs coated with antimicrobial metabolites of *T. viride* were more potent than their chemical counterpart in killing pathogenic bacteria. These results clearly demonstrated the simultaneous synergistic activity of secondary metabolites and proteins of *T. viride* with silver NPs in enhancing antibacterial activity compared with the partial inhibition caused by CSNPs coated with citrate [63].

In related research, broad-spectrum antifungal activity was demonstrated for biogenic silver (BS) NPs synthesized from the cell-free filtrate of *T. viride* (MTCC 5661). Compared with chemically synthesized silver NPs (CSNPs) of similar shape and size, antifungal activity was higher in BSNPs in comparison with CSNPs; BSNPs also increased the inhibition of mycelial growth and weight reduction of the fungal pathogens *F. oxysporum* and *Alternaria brassicicola* by 20–48.8%. These results provide us with an understanding of the role of antimicrobial metabolites produced by *Trichoderma viride* in coating BSNPs to enhance their antimicrobial properties against the plant pathogen *A. brassicicola* [64].

Biological control agents are known to exhibit different antagonistic activities against pathogens due to the diversity of the active secondary metabolites they secrete. Therefore, NPs capped with more active secondary metabolites may have increased activity against pathogens. For example, copper and silica NPs synthesized from the cell-free culture filtrate of *Trichoderma atroviride* showed high inhibition against the growth of the fungal plant pathogens *Poria hypolateritia* and *P. theae*, compared with similar shaped and sized NPs prepared from both *Streptomyces griseus* and *Pseudomonas fluorescens* [65]. There is additional evidence for the participation of the active compounds secreted of *Trichoderma* in the synthesis of NPs from a study conducted by Tomah [28], in which AgNPs were synthesized using the cell-free culture supernatant of *T. virens* HZA14. Through EDS data analysis of AgNP powder, it was noted that the relative proportion (1.24%) of the sulfur element was higher than that (1.04%) of the nitrogen element. Also, the FTIR spectra revealed the presence and binding of protein, carbohydrates, heterocyclic compounds, and fatty acids with AgNPs. However, a peak with a larger shift change revealed that the AgNPs bound strongly with oxygen from oxidized or reduced forms of gliotoxin. Therefore, the authors have suggested two interaction patterns with AgNPs: binding with negatively charged carboxyl groups or with dithiol groups in gliotoxin. On the other hand, AgNPs capped by gliotoxin exhibited high inhibitory activity against hyphal growth, sclerotial formation, and myceliogenic germination of the sclerotia of *Sclerotinia sclerotium*. Capping of the AgNPs by active compounds derived from *T. harzianum* enabled the control of the plant pathogen *S. sclerotiorum*, decreased genotoxicity toward the HaCaT cell line, and did not inhibit non-target microorganisms of agricultural importance compared with the commercial silver AgNP-C [66].

**Figure 2:**
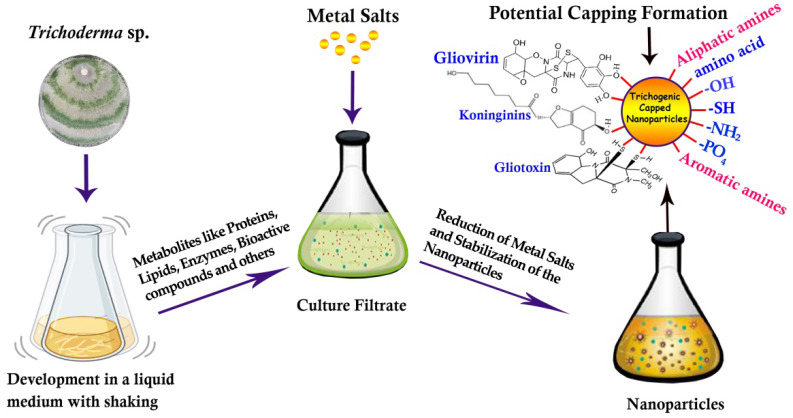
The proposed scheme of the synthesis of NPs obtained by biomolecules released by the *Trichoderma* sp. in the filtrate that are responsible for the capping and formation of NPs.

According to the scheme that was proposed by Bilesky-José [67], the synthesis and stability of iron oxide (Fe) NPs occurred by hydrolysis and by using biomolecules released by *T. harzianum* in the filtrate, such as proteases, chitinases, glucanases, peroxidases, phosphatases, auxins, hydrolytic enzymes, siderophores, glyoxin, viridin, trichodermin, and suzucacillin. The FeNP has high biological activity against *S. sclerotiorum* mycelial growth and the formation of new sclerotia; as noted, *T. harzianum* alone also led to inhibition of white mold mycelial growth and the formation of new sclerotia. As proof of concept, the FeNP activity in controlling a white mold pathogen occurs because of the synergy with biomolecules of *Trichoderma* responsible for capping off the NPs in the synthesis process. To date, NPs using *Trichoderma* culture filtrates have been synthesized extracellularly in vitro by the stimulation of 10 g of mycelia (fresh weight) of *Trichoderma* fungus to secrete the active metabolites in double-distilled water after incubating in a rotating shaker at 25 °C and 150 rpm for 96 h. Whatman No. 1 filter paper is used to separate and purify the fungal filtrate and then the pH is adjusted to 7.0. One hundred milliliters of the fungal extract product is mixed with 1 mM of the required metal in solution, and the reaction mixture is incubated in a rotating shaker at 25 °C and 150 rpm in dark conditions for 96 h. The color change of the reaction liquid could indicate the presence of NPs [28]. The scheme proposed for the synthesis of nanoproducts extracellularly by using secreted products of *Trichoderma* is shown in Figure 2. Through the scientific extrapolation of the mentioned literature, and through noting the role of these metabolites in increasing the NPs properties toward plant pathogens, we can assume that active metabolites secreted from the biocontrol agent *Trichoderma* are responsible for capping off the NPs in the process of synthesis and provide more stable and safe nanomaterials in a sustainable way.

## 4. Possible Modes of Action

Numerous studies have shown that biosynthesized NPs have the potential to suppress several plant pathogens. However, the exact mode of action of NPs is still not completely understood [68]. The reports indicated some of the mechanisms of action pursued by NPs in their antagonistic activity, such as protein disarrangement [68], production of reactive oxygen species (ROS), antioxidant degradation of the ROS, disruption, imbalance, lack of permeability in membranes, genotoxicity, and transporter gene expression inhibition [69].

The initial effect of NPs on live microbial cells is the direct physical interaction between them, which is called the adhesion process. The adhesion process occurs due to the electrostatic interaction between the positive surface charge of the NPs and the negative charge of the fungal cell wall, which is mediated by glycoproteins [70]. After the initial direct contact, cellular uptake of NPs occurs via direct physical interactions and ROS accumulation. Nanoparticle penetration inside the cell is dependent on the structure and engineering of the fungal cell wall; generally, most of the hyphae cell walls comprise a mixture of chitin, β-1,3 glucans, and β-1,6 glucans and a wide variety of glycoproteins [71,72]. Certain glycoproteins, known as adhesins, participate in the adhesion to organic and inorganic surfaces and are involved in host–pathogen interactions [73]. The nanomaterial penetration into the fungal cell occurs by one of two possible mechanisms; direct internalization of NPs in the cell wall, especially of small-sized spherical NPs, and the internalization of the NPs by a specific receptor or through membrane-spanning ion-transport proteins [74]. The effect of the NPs on the fungi cell wall may be through the action of ROS generated by the NPs and/or Zn^2+^ produced by the dissolution of the NPs in the culture medium by the enzymes N-acetylglucosamine and β-1,3-D-glucan synthase, as these enzymes are known to be involved in the syntheses of chitin and β-1,3-D-glucan, respectively, which are important components in the structure of the fungi cell wall. Inhibition of these enzymes may lead to some abnormalities in the cell wall, such as thickening of the cell wall and liquefaction of the cytoplasmic contents [75].

The OH radicals generated by the NPs may cleave the N-acetylglucosamine monomer links of the chitin or/and D-glucose monomer links of the β-glucans by replacing the H atom at position 1, and the glycosidic bonds are converted to ester bonds, resulting in the loosening of the cell wall structure, which allows intracellular material such as ions, proteins, or cellular energy reservoir (ATP) to pass through the cell boundaries, leading to the death of the cell [76]. At the molecular level, NPs and released ions disrupt electron transport and protein oxidation and affect the potential of the mitochondrial membrane by increasing the levels of transcription of genes in response to oxidative stress. This induces the generation of reactive oxygen species (ROS), triggering oxidation reactions catalyzed by the different metallic NPs, causing severe damage to proteins, membranes, and deoxyribonucleic acid (DNA), and interfering with nutrient absorption. The ions of the charged TiO_2_NPs result in a decrease in the genes of antioxidant enzymes in human glial cells in vitro [77,78].

Similar to this, some of the *Trichoderma* species applied in biological control release enzymes and metabolites that degrade the cell walls of pathogenic fungi [79]. Similarly, a *T. pseudokoningii* peptaibol, called trichokonin VI, is known to form voltage-gated channels in the membrane, and it ultimately induces programmed cell death in *F. oxysporum* [80]. Also, it was noted that all the concentrations of secondary metabolites of *T. viride* used completely damaged the DNA of *Macrophomina phaseolina* [81]. A study suggested that the primary mechanism of action of gliotoxin extracted from *T. virens* involves the selective binding to cytoplasmic membrane thiol groups [82], causing cytoplasmic leakage in cells of *R. solani* [83], and the addition of exogenous gliotoxin to the sclerotia led to degradation of the sclerotia of *S. sclerotiorum* [84]. Also, gliotoxin treatment caused marked alteration of the hyphal cells of *S. rolfsii*, like a reduction in the number and length of mitochondrial cristae and striking plasmolysis, and ultrastructural changes were induced [85]. Gliotoxin can induce the production of ROS via intracellular redox cycling [86]. By comparison, AgNPs produced by the *Rhizopus arrhizus*, *T. gamsii*, and *A. niger* strains were found to be active against *E. coli*, *S. aureus*, and *P. aeruginosa* but to different extents depending on the structure of the silver crystal which was affected by the chemical environment of the fungi linked to the nitrate reductase activity profiles [87]. *Trichoderma*, as fungi, can accumulate metals by the sequential action of reductase enzymes such as NADPH-dependent nitrate reductase, leading to the reduction of metal salts and the final production of metal NPs [88].

In this review, we describe the mechanisms of NPs synthesized by *Trichoderma* derivatives and the potential synergistic support for *Trichoderma*-secreted secondary metabolites involved in particle synthesis for plant–pathogen inhibition. The action mechanism of AgNPs enhanced by *Trichoderma* exometabolites was summarized by Hirpara and Gajera [89], who reported the increased production of free radicals leading to disrupted permeability of the outer wall of fungi cells, resulting in leakage of cellular proteins and sugars, in addition to the inactivation of respiratory chain dehydrogenase by entry into the inner membrane and inhibition of cell respiration in the phytopathogenic *Sclerotium rolfsii*. In a more accurate description of the mode of action of NPs, the biosynthesized silver nanoparticle (BSNP) by *T. viride* had superior antifungal activity than its chemically synthesized silver CSNP, where biosynthesized silver (BS) NPs led to a reduction in dry weight, cell wall disruption, complete loss of membrane integrity, and imbalance in osmotic pressure leading to cell death of the fungal pathogens *F. oxysporum* and *A. brassicicola* in comparison with their chemically synthesized (CS) counterpart NPs [64]. Generated ROS and superoxide radicals were higher in BSNP- than CSNP-treated cells. The expression of oxidative enzymes failing to cope with oxidative stress decreased in BSNP-treated fungal cells in comparison with CSNP treatment. The BSNP treatment completely disrupted the inbuilt redox homeostasis of the pathogen, while CSNP treatment failed to do so. This study suggested that the amalgamation of antifungal properties of silver NPs and the cell-free culture filtrate of *T. viride* in BSNPs enhance the desired effects against fungal plant pathogens.

It was suggested that ZnO NPs formed by *T. asperellum* might successfully permeate pathogenic microorganism cell membranes through the lipid bilayer because of their reduced hydrophobicity and absence of surface charge. Indeed, the ZnO NPs showed obvious destruction of the cell walls and plasmolysis of the internal organs of *R. solani* (RS9), *Fusarium* sp. (F10), and *M. phaseolina* (M4) [90]. The SEM micrographs assisted in elucidating the mechanism/relationship between the bacteria and the ZnO NPs biosynthesized by *T. asperellum* and their antibacterial activity. It is obvious that the ZnO NPs initially adhered to the outer cell membrane of *S. aureus* and then penetrated the cell entirely, perhaps resulting in cell death. The use of SEM to visualize the bacterial biofilms revealed a wide range of morphological changes in the biofilm topologies. There were remarkably fewer dispersed cell aggregates and fewer viable cells in the aggregates in the biofilms after 24 h of exposure to the ZnO NPs. These findings show that the ZnO NPs were more efficient than tetracycline in inhibiting biofilm development and in disrupting the preformed biofilms of *S. aureus* [91].

**Figure 3:**
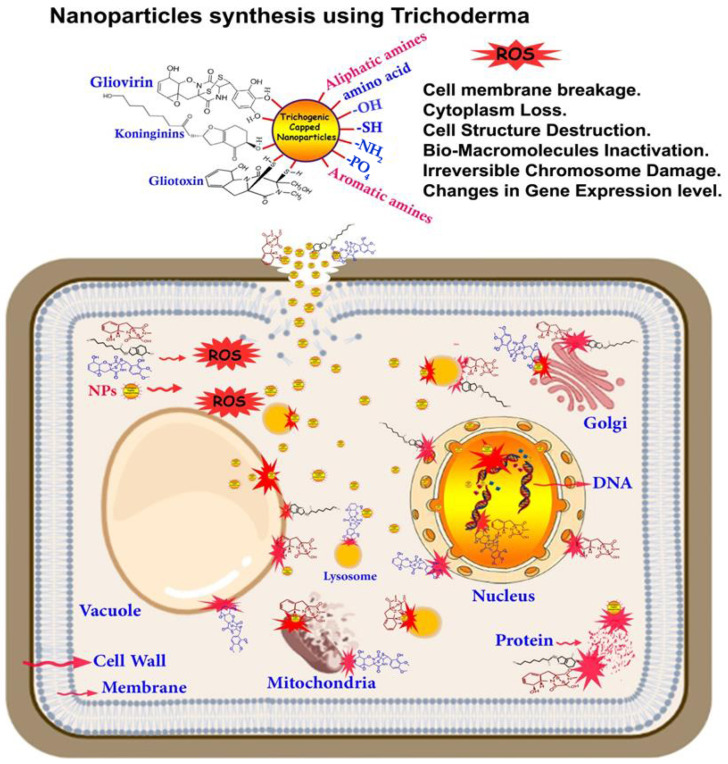
The potential schematic illustration of antifungal activity for NPs synthesized using the bioactive compounds from the metabolism of *Trichoderma*; mechanisms of action of NPs are doubled through synergism with bioactive compounds that leads to an increase in damage in the walls and organelles and, finally, cell death.

The morphological changes to the sclerotia of the pathogen *S. sclerotiorum* treated with AgNPs synthesized using a cell-free aqueous filtrate of *T. virens* HZA14 producing gliotoxin were revealed by Tomah [28]. The mechanism of action pattern of AgNPs had been recognized by both EDS analysis and SEM micrographs, which revealed the adhesion and accumulation of Ag and the production of lamellar fragments. The O, N, C, and S elements were also revealed on hyphae cell surfaces of the sclerotia, which may belong to the gliotoxin compound secreted from *T. virens*. The biological efficiency of NPs covered with *Trichoderma* bio-agent derivatives compared with synthetic or uncapped NPs gives us a clear and understandable picture of the role played by the effective metabolites that act as a capping agent on the one hand and a synergistic agent with NPs with their antimicrobial activity on the other hand. From here, we can support the theory that “the type of capping and its source significantly influences the biological functions of NPs”, and we conclude that the bonds formed between the NPs and the metabolites evoked by *Trichoderma* lead to an improvement in their biological functions and double their capabilities to damage and degrade the walls and organelles of cells, which lead to the death of plant pathogens, as shown in the proposed schematic illustration in Figure 3.

## 5. Applications of Nanoparticles Synthesized by *Trichoderma* spp.

To improve and develop this review, a survey of literature published only on the synthesis of NPs by *Trichoderma* species from 2008 to 2021 was performed using three databases: Google Scholar (https://scholar.google.com, accessed on 25 June 2022), Research Gate (www.researchgate.net, accessed on 25 June 2022), and Academia (www.academia.edu, accessed on 25 June 2022). The search approaches were centered on all *Trichoderma* species used in the synthesis of NPs.

### 5.1. In Medicine and Healthcare

In recent years, nanotechnology-based therapeutic and diagnostic approaches have shown promising eco-friendly potential in medical and pharmaceutical applications. *Trichoderma* spp. contributed to the biosynthesis of NPs, and these showed anti-cancer activity when tested on different human cancer cell lines. As an advanced step in synergy, the AgNPs synthesized using extracellular filtrates of *T. viride* showed synergistic activity with antibacterial compounds such as ampicillin, kanamycin, erythromycin, and chloramphenicol, whose inhibitory activity was increased in the presence of AgNPs against the tested Gram-negative strains *S. typhi* and *E. coli* and the Gram-positive strains *S. aureus* and *M. luteus* [92]. As a new methodology, a fungal *T. viride* cell filtrate was used in the biosynthesis of gold NPs in synergy with vancomycin to produce VBGNPs, thus restoring antibacterial activity against vancomycin-resistant *S. aureus* (VRSA); the reason for this is that VBGNPs non-specifically bind to transpeptidase instead of terminal peptides of the glycopeptidyl precursors on the cell surface of VRSA, and therefore these VBGNPs effectively lyse the cell walls of VRSA [93]. The biosynthesis of AgNPs using the extracellular filtrate of *T. harzianum* is considered more efficient than from other fungi and is not harmful to humans, is cost-effective, and can be applied in the integrated control program against mosquitoes for public health [94]. The AgNPs synthesized by cell filtrates of *T. inhamatum* incubated with AgNO_3_ exhibited antibacterial activity against the human-disease-causing bacteria *E. aerogenes*, *S. typhimurium*, *S. aureus*, and *S. pyogenes*, as tested by the agar well diffusion method [36]. In the pharmaceutical field, NPs can be used to replace antibiotics and/or and for drug delivery. The AgNPs biosynthesized using *T. viride* cell extracts showed greater antibiotic activity against Gram-negative bacteria (*Shigella boydii*, *S. sonnei*, *Acinetobacter baumannii* and *S. typhimurium*) than against Gram-positive bacterium (MRSA); this could because of the presence of the peptidoglycan layer in the Gram-negative bacterial cell walls [95].

Moreover, necrosis of human pulmonary carcinoma A549 cells was observed when these were treated with ZnNPs synthesized using the fungal mycelial water extract derived from the *T. harzianum* strain SKCGW009. Light and fluorescent microscopic examination confirmed their promising activity in inhibiting human lung carcinoma [43]. The filtrate of *T. viride* exhibited the ability to reduce Ag ions and the biosynthesis of SNPs and had powerful cytotoxicity against the Hep-2C cell line and the RD cell line; in addition, it had immune-stimulating potential by increasing the production of IgA and IgM [96]. Hence, using NPs synthesized by *Trichoderma* spp. is a further step toward eco-friendly synthesis with applicability in pharmaceutical formulations as an antimicrobial [97]. The AuNPs were prepared using the crude protein fungal extract of the *T. harzianum* strain SKCGW009 and mixed with an aptamer (APT) to obtain APT-FE-AuNPs. Their toxicity was tested in normal and cancerous cells, and the results showed that the synthesized NPs exhibited less cytotoxicity in normal NIH3T3 cells while the APTFE-AuNPs (λmax 521 nm) showed significant cytotoxicity in A549 and LN229 cells [98]. Broad-spectrum antibacterial activity against two Gram-positive bacteria (*S. aureus* and *B. subtilis*) and two Gram-negative bacteria (*E. coli* and *R. solanacearum*) was achieved using AgNPs synthesized using secondary metabolites secreted by *T. harzianum* [99].

### 5.2. In Agriculture

In addition to the successful large-scale use of *Trichoderma* spp. in agricultural applications [100], these species also play a distinct role in the biosynthesis of NPs, enabling environmentally friendly agricultural practices in various fields such as stimulating plant growth, improving the composting process, and controlling different plant diseases. The AgNPs were synthesized using culture filtrates of the fungus *T. asperellum* and its physiological effects were evaluated after foliar spraying on tea; the results showed that AgNPs induced the plants to increase their chlorophyll content, moisture content, relative water content, total soluble sugar, total protein, and lipid peroxidation [101]. For the increased viability of oilseeds, the use of AgNPs synthesized using cell-free filtrates of *T. harzianum* showed an increase in the germination percentage for both Sunflower (*Helianthus annuus*) and Soybean (*Glycine max*) seeds with an increase in soaking time with the AgNP solution [102]. The AgNPs and TiO_2_NPs were synthesized using *T. citrinoviride* extracts and studied as seed priming agents to promote the seed germination and seedling growth of *Solanum lycopersicum*. The study revealed that lower concentrations of TiO_2_NPs (25 and 50 μg/mL) had a positive effect on seed germination and seedling vigor while the AgNPs showed an increase in the activities of antioxidant enzymes such as catalase, superoxide dismutase, and peroxidase [103]. The use of the *Trichoderma* species to synthesize metal NPs is a favored method in the control of agricultural pests because of their strong activity against pests and because resistance against them has not yet been induced. In the vegetable and fruit preservation field, the AgNPs biosynthesized using the filtrate of *T. viride* and their incorporation into sodium alginate was evaluated, where the thin film of AgNPs incorporated with sodium alginate led to the protection of carrot and pear from human-pathogenic bacteria and increased their shelf life with respect to weight loss and soluble protein content [104]. The TDNPs were extracellular synthesized from *T. viride* and applied as a novel biopesticide; these produced high mortality in larvae of *Helicoverpa armigera* while no toxic effects were found in the earthworms *Eudrilus eugeniae* in artificial soil assays when treated with TDNPs at 100 ppm [105].

To control plant pathogens in modern ways that are more efficient than traditional agricultural methods and which have less impact on the ecosystem, unlike industrial pesticides, researchers have tended to develop methods of producing NPs as alternative products that are efficient at reducing the spread of plant diseases, environmentally acceptable, and lower cost. Using AgNPs synthesized using filtrates of both *T. citrinoviride* and *T. velutinous*, a high inhibition ratio was obtained against the fungus *F. oxysporum* [106], which is considered one of the more difficult causes of plant disease to manage. A concentration of 200 ppm of both biogenically synthesized AgNPs and AuNPs using culture filtrates of *T. asperellum* showed that the highest inhibition in radial growth of *R. solani* was 73.39% for the AgNPs, while inhibition by AuNPs at the same concentration was 60.83%. In the pots experiment, AgNPs at 200 ppm showed increased plant growth parameters and a reduced percent disease incidence of sheath blight in rice (20.00%) compared with the inoculated *R. solani* control (88.00%); they also increased the concentration of vital secondary metabolites like phenols, flavonoids, terpenoids, and total soluble sugars [107].

The bird eyespot disease in tea leaves caused by *Cercospora theae* was controlled through foliar spraying of silver (Ag) NPs and copper (Cu) NPs synthesized by three antagonists, one of which is *T. atroviride* [108]. The development of nanofibrous mats is obtained by keeping the mixture of the chitosan with *T. viride* at 28 °C, which leads to the formation of nanofibrous mats that grow much faster and that fight for space and nutrients with the fungi *Alternaria* sp. and *Fusarium* sp. [109]. The biosynthesis of silver (Ag) NPs mediated by four species of *Trichoderma* (*T. viride*, *T. hamatum*, *T. harzianum*, and *T. koningii*) was achieved and showed high inhibition levels against four soil-borne *Fusarium* spp. fungi, *F. solani*, *F. semitectum*, *F. oxysporum* and *F. roseum*, in vitro [110]. Green synthesis of AgNPs by using cell-free culture filtrate of the biocontrol strain *T. harzianum* Th3 exhibited effective mycelial growth control, reaching 65, 62, and 59% of the root rot pathogens of groundnut, viz., *A. niger*, *S. rolfsii*, and *M. phaseolina*, respectively [111].

Environmentally friendly AgNPs were rapidly synthesized from the supernatant of *T. asperellum* Q1 and exhibited high antifungal activity using the agar well diffusion method against *F. oxysporum* f. sp. *conglutinans*, *F. oxysporum* f. sp. *cucumerinum*, *F. oxysporum* f. sp. *niveum*, *F. oxysporum* f. sp. *vasinfectum*, and, *F. graminearum*, comparing both cell-free supernatant and silver nitrate solution. The green synthesis of α-Fe_2_O_3_ NPs mediated by *T. harzianum* contributed to seed germination due to being from micronutrients of plants and working as an antifungal in inhibiting the pathogen *S. sclerotiorum* (white mold) in vitro [67]. Applications of NPs synthesized using *Trichoderma* spp. in plant disease control include the controlled release of nano-pesticides through seed soaking, plant watering, and, subsequently, foliar spray [112] or the moistening of leaves. Different amounts of AgNPs were successfully biosynthesized using the culture liquid and culture water from different *Trichoderma* strains, viz., *T. atroviride*, *T. crissum*, *T. longibrachiatum*, *T. spirale*, *T. virens*, *T. afroharzianum*, *T. koningiopsis*, *T. hamatum*, *T. citrinoviride*, and *T. velutinum*, with higher antibacterial activity against *Enterococcus pernyi* that causes oak silkworm empty-gut disease but was not toxic to larvae and could improve the growth and development of larvae [113]. Table 1 shows the results of a survey of some scientific articles on the biosynthesis NPs using derivatives of *Trichoderma* spp. and their activity assessment in vitro.

### 5.3. In Bioremedies for the Environmen

The environment (water and soil compartments) is exposed to numerous industrial pollutants, and their removal requires physical and chemical techniques [114]. Conventional physical and chemical methods for pollutant disposal encompass various approaches, including chemical reduction, electrochemical treatment, ion exchange, sedimentation, and evaporation recovery [115,116]. The conventional methods of NP synthesis have several drawbacks, including the generation of other toxic byproducts, the destruction of organic compounds, chemical consumption, high cost of recovery, incomplete removal, and high energy consumption [116]. Therefore, there is an urgent need to find an alternative technology for nanoparticle synthesis that is safe, efficient, and environmentally friendly. Recently, different producing roles for NPs in bioremediation have been widely studied due to their larger surface area and small size, as they can therefore either act as catalysts or adsorb the pollutants over their larger surface area. The unique properties of the *Trichoderma* species, such as their metabolic diversity, tolerance of environmental contaminants, and detoxification ability, make them a suitable and persuasive tool in NP synthesis and for their application in diverse fields such as coating, packing, biotechnology, industry, and environment. The contribution of gold (Au) NPs synthesized by derivatives of the *T. harzianum* was highlighted to be a prominent candidate in the ultra-sensitive detection of mercury in both pure water samples and in samples barbed with other ions [117], the bioremediation of wastewater for the removal and reduction of copper ions in impacted local regions [118], and to have photo-catalytic potential in the degradation of methylene blue using cadmium sulfide (CdS) NPs formed using the fungal biomass of *T. harzianum* [44]. The disposal of dye-containing effluents without serious damage to the environment by the decolorization of various azo dyes was efficiently catalyzed using gold NPs (AuNPs) synthesized by a strain of the *Trichoderma* sp., WL-Go [39]. Biosynthesized gold NPs from a cell-free extract of the *T. viride* exhibited activity as an efficient biocatalyst that reduced 4-nitrophenol to 4-aminophenol in the presence of NaBH_4_ [38]. Use of the biogenic Au NPs and Ag NPs synthesized by *T. hamatum* cell filtrates as catalysts for bioelectricity production in a microbial fuel cell (MFC) approach under anaerobic conditions was eco-friendly, economically feasible, and facilitated electricity production [119]. On the other hand, most fungi, when exposed to toxic stress resulting from pollution, secrete biologically active molecules as a defensive response, which leads to the reduction, precipitation, stabilization, and modification of the shape of the ions and then their linkage to biological molecules [120,121]. The fungal response leads to the reduction and deposition of metals in the form of NPs that form inside or outside the fungal cells and clean the surrounding ecosystem of toxic metals [122,123]. Recently, some *Trichoderma* strains have been demonstrated to have the ability to tolerate high stress and to be resistant to a range of toxicants like cyanide (CN), heavy metals, and polyphenolics [124]. When some strains of *Trichoderma* such as the *Trichoderma* strain T32, a mutant of pesticide-tolerant *Trichoderma*, were applied to an agrochemical-contaminated site, they possessed extensive potential to degrade fungicides such as Carbendazim [125]. Hence, they have been successfully used in the biological treatment of a polluted environment through their ability to reduce some metals such as copper [118]. For the biosorption of gold, the fungal biomass of *T. harzianum* showed a gold biological sorption capacity of 1340 mg/g^−1^ and a removal rate of 62%, while promoting the biogenic synthesis of NPs simultaneously [126]. Recently, the adsorption capacity of Pb by silica oxide (SiO_2_) NPs biosynthesized by *T. harzianum* MF780864 in the Nile tilapia fish (*Oreochromis niloticus*) aquaria was evaluated by testing different concentrations of SiO_2_NPs, wherein 1 mg/L exhibited the highest Pb adsorption efficiency, a significant increase in growth and hematological parameters, an enhancement of antioxidant capacity (TAC), an increase in the immune-related gene expression of IL-1b, and a noticeable decrease in Pb residue levels in fish muscles in the SiO_2_NP-treated groups [127]. For the first time, biogenic Mt NPs formed using *T. guizhouense* NJAU4742 and magnetite (Mt) was shown to exhibit intrinsic peroxidase-like activity [128].

**Table 1:** Some of the Trichogenic applications in medicine and healthcare, agriculture, and environmental bioremediation.

*Trichoderma* spp.	Localization	Nanoparticles			Application and Effect	Reference
		NPs	Size (nm)	Shape		
*T. koningii*	Mycelial biomass	Ag	8–24	Spherical	Antibacterial toward human pathogens	[129]
*T. harzianum*	Cell-free filtrate	Ag	4.66	Spherical	Antifasciolasis toward human pathogens	[130]
*T. koningii*	Cell-free filtrate	Au	10–14	Spherical	Anticancer in humans	[131]
*T. viride*	Cell-free filtrate	Ag	28	Bowl-Shaped	Antibacterial toward human pathogens	[132]
*T. harzianum*	Cell-free filtrate	Ag	10–20	Crystalline	Larvicidal and pupicidal in plants	[94]
*T. atroviride*	Culture filtrates	Ag	14–21	Hexagonal	Larvicidal in plants	[133]
*T. viride*	Cell-free filtrate	Ag	4–16	Spherical and Oval	Antimicrobial toward human pathogens	[134]
*T. viride*	Culture filtrate	Mg	45–95	Crystalline	Antibacterial toward human pathogens	[41]
*T. harzianum.*	Mycelial biomass	Ag	5–29	Round and Oval	Antimicrobial toward human pathogens	[135]
*T. gamsii*	Cell-free filtrate	Ag	63.80	Spherical	Antibacterial toward human pathogens	[87]
*T. harzianum.*	Mycelial biomass	Au	26–34	Spherical	Antibacterial toward human pathogens	[136]
*T. asperellum*	Cell-free filtrate	Cu	110	Spherical	Anticancer in humans	[43]
*T. hamatum*	Cell-free filtrate	Au	5–30	Spherical and Pentagonal	Antibacterial toward human pathogens	[40]
*T. citrinoviride*	Mycelial cell lysate	Ti	25–200	Pentagonal and Triangular	Antibacterial toward human pathogens	[45]
*T. longibrachiatum*	Cell-free filtrate	Ag	10	Monodispersed and Spherical	Antifungal toward phytopathogens	[61]
*T. atroviride*	Mycelial biomass	Ag Au	10–50 50–75	Spherical and Triangular	Antifungal toward phytopathogens	[137]
*T. harzianum*	Cell-free filtrate	Cu	38–77 W 135–320 L	Elongated Fibres	Antifungal toward phytopathogens	[138]
		Zn	27–40 W 134–200 L	Fan and Bouquet Structure		
*T. harzianum*	Cell-free filtrate	Ag	12.7 ± 0.8	Roughly Spherical	Antifungal toward phytopathogens	[139]
*T. virens*	Cell-free filtrate	Ag	5–50	Spherical to Oval	Antifungal toward phytopathogens	[28]
*T. hamatum* *T. harzianum*	Mycelial biomass	Si Ti	5–26 2–52	Spherical	Antifungal toward phytopathogens	[112]
*T. harzianum*	Cell-free filtrate	Ag	20–30	Spherical	Antifungal of Phytopathogenic	[140]
*T. harzianum* *T. reesei*	Fungal extract	Zn	12–35	Different morphology	Antibacterial toward phytopathogens	[141]
*T. harzianum*	Cell-free filtrate	Fe Zn	20–60 10–40	Spherical	Antifungal toward phytopathogens	[142]
*T. viride*	Cell-free filtrate	Ag	100–250	Spherical and Irregular-like	Antifungal toward phytopathogens	[143]
*T. harzianum*	Mycelial extract	Ag	3–20	Monodispersed Round	Antibacterial toward phytopathogens	[144]
*T. interfusant*	Culture filtrate	Ag	59.7–62.6	Spherical	Antifungal toward phytopathogens	[89]
*T. harzianum*	Culture filtrate	Se	40–60	Spherical	Antifungal toward phytopathogens	[145]
*T. atroviride*	Cell-free filtrate	Cu Si	5–25 12–22	Spherical Spherical	Antifungal toward phytopathogens	[65]
*T. harzianum*	Mycelial biomass	Au	26–34	Spherical	Bioremediation	[117]
*T. koningiopsis*	Extracellular	Cu	87.5	Spherical	Bioremediation of wastewater	[118]
*T. asperellum*	Mycelial biomass	Au	20–180	Spherical and Triangular	Decolorization of azo dyes	[39]
*T. viride*	Cell-free filtrate	Ag	2–4	Spherical	Biosensor and Bioimaging	[146]
*T. harzianum*	Mycelial biomass	Au	15	Spherical	Biosorption of gold	[126]
*T. harzianum*	Mycelial biomass	Cd	3–8	Spherical	Decolorization	[44]
*T. hamatum*	Cell-free filtrate	Ag Au	50–150	Spherical and Cubical	Bioelectricity	[119]
*T. viride*	Cell-free filtrate	Au	20–120	Spherical	Decolorization	[38]
*T. atroviride*	Culture filtrate	Se	60.48–123.16	Spherical	Antifungal toward phytopathogens	[147]
*T. asperellum*	Culture filtrate	Se	49.5	Irregular	Antifungal toward phytopathogens	[31]
*T. atroviride*	Cell-free filtrate	Ag	10–15	Spherical	Antifungal toward phytopathogens	[148]
*T. harzianum*	Mycelial biomass	Ag	7.8	Spherical	Antifungal toward phytopathogens	[149]

## 6. Nanoparticle Toxicity

Biogenic NPs are considered a promising alternative to their synthetic versions. However, the environmental impact of such nanomaterials is still scarcely understood. Mycogenic Ag NPs induce adverse effects on organisms at different trophic levels. Thus, a more careful assessment of their industrial application, economic feasibility, and ecotoxicological impacts is crucial [150,151]. Generally, NPs manufactured from the derivatives of the non-pathogenic fungus *Trichoderma* spp. can be considered an ideal alternative for many applications, especially those that have direct contact with health, the environment, and food, due to their several advantages. Toxicity levels of biogenic AgNPs can vary according to the use of reducing agents and biological stabilizers that result in different coatings [152]. To examine toxicity, the effect of Ag NPs synthesized by *T. harzianum* on the *Allium cepa* chromosomes were assessed [140]; Ag NPs at different concentrations induced changes in the mitotic index and altered the index of onion cells by lower values than AgNPs synthesized using the fungus *F. oxysporum* [153], and these were significantly lower than AgNPs synthesized using the male inflorescence of the plant *Pandanus odorifer* [154], which has lower values than those obtained with commercial NPs (Sigma-Aldrich, St. Louis, MO, USA, size < 100 nm, purity 95.5%). Furthermore, the AgNPs synthesized using *T. harzianum* presented low levels of cytotoxicity and genotoxicity toward the V79, 3T3, and HaCaT cell lines [60]. Hence, the safety evaluation demonstrated that the functional TSNPs synthesized by culture filtrates of *T. harzianum* were not toxic to human hepatic stellate cell line LX-2, the intestinal epithelial cell line HIEC, and the gastric epithelial cell line GES-1 at 50 μg/mL [145]. It was confirmed that there was no toxicity from nano-copper and nano-silica formed by the mycelial-free filtrate of *T. atroviride* when exposed at 0.5, 3, and 30 mg concentrations in the zebrafish in terms of the viability percentage of an embryo, hatching rate, body mass index, and heartbeat count [65]. Laboratory studies showed the cytotoxic effect of biologically synthesized silver NPs against MCF-7 cancer cell line proliferation in a time and concentration-dependent manner by the MTT assay, suggesting that Ag NP synthesis by filtrates of *T. viride* might be used to treat breast cancer [155]. The α-Fe_2_O_3_ NPs mediated by *T. harzianum* were noted to not show cytotoxicity in different cell lines, compared with the controls, and did not lead to changes in the mitotic index in *A. cepa* cells at the concentrations used [67]. However, the slight or no toxicity at low concentrations of the NPs formed by the *Trichoderma* spp. mentioned in the above studies were measured in tests at the in vitro level that differ radically from the in vivo condition. Therefore, it is necessary to conduct tests under in vivo conditions to obtain an accurate assessment of the toxicity of these particles to humans and plants. On the other hand, the production of biogenic titanium NPs using *T. harzianum* as a reducing and stabilizing agent (NPTiOIVR-NS) was successful. NPTiOIVR-NS did not induce cytotoxicity in the tested cell lines, even at the highest concentrations and with exposure to ultraviolet radiation. Regarding the effects on microorganisms of agricultural importance, no inhibition was observed on *B. thuringiensis*, *P. aeruginosa*, *Bradyrhizobium japonicum*, and *Beauveria bassiana*, and only minor alterations were observed in the quantification and proportion of bacterial genes involved in the soil nitrogen cycle. This could be related to the presence of capping from the *Trichoderma* employed in the synthesis. Morphological changes and oxidative stress were not observed in soybean plants cultivated in the soil exposed to the NPs [156].

## 7. Advantages and Challenges

Most *Trichoderma* species have many advantages that make them shine in different scientific fields. There are several advantages specifically related to the *Trichoderma* fungus; advantages that are more important are that they are non-pathogenic toward humans and plants, can be easily isolated and cultured, rapidly grow on diverse inexpensive organic substrates like the media of potatoes, and have the capacity to produce a wide range of enzymes and secondary metabolites [6,7]. With regard to advantages related to NPs produced by *Trichoderma* derivatives, the NPs biosynthesized using *Trichoderma* species have additional benefits, for example, they can be produced rapidly, use a cost-effective method that is clean, eco-friendly, easy to cultivate in high amounts of fungal biomass, and thus can produce a high number of NPs [97,157] that are stable for a long time and do not show a significant accumulation after storage for more than 6 months. In addition to their controlled size and morphology [33], they have an impact against a large number of drug-resistant pathogenic bacteria [158]. Another important issue is related to the size and shape of NPs; most particles synthesized by derivatives of *Trichoderma* strains that are used as biological control agents are NPs with small sizes of than 100 nm, and most are spherical (spherical shapes account for 73% of all shapes). In this regard, some studies show that the antibacterial effect of nano-silver depends strongly on the size and shape of the particles [159].

The spherical Ag NPs were more stable and demonstrated better antibacterial activity against bacterial strains compared with the triangular and rod-shaped AgNPs [160,161]. These features have made the *Trichoderma* species a leading natural resource compared with other fungal species to be exploited in the synthesis of NPs. However, the use of *Trichoderma* in the synthesis NPs still has some challenges:

1. Not all *Trichoderma* strains can synthesize NPs, so the process of obtaining a suitable isolate requires the testing of many *Trichoderma* isolates, which takes time and effort.
2. Studying is required to know the growth conditions of the strain used and the impacting conditions, such as temperature, pH, shaking, and the time required to secrete secondary metabolites.
3. The metabolites vary between *Trichoderma* isolates of the same species as well as between isolates of different species, so for positive results and NP formation, it is still necessary to know the organic compounds that contribute to the synthesis of the nanomaterial, which requires further tests such as FTIR and GC Mass Spectometry.

## 8. Future Developments

Selenium (Se) is an important micronutrient required by both plants and animals but must be taken at low concentrations. It is currently considered a naturally occurring mineral in the soil and is absorbed and accumulated by plants, thereby entering the food chain and contributing to protecting the plants from a variety of abiotic stresses [162]. In animals, Se acts as an antioxidant and helps in reproduction, immune responses, and thyroid hormone metabolism [163]. *Trichoderma*-derived NPs can be synthesized with a wide range of particle size distributions. There are advantages of reproducibility as well as control over particle size, shape, and dispersion. As a result, in future efforts, the development of innovative nano-fungicides, nano-fertilizers, and nano-sorbents generated by *Trichoderma* and the evaluation of these nano-formulations for the remediation of heavy-metal-contaminated industrial wastewater should be given increased attention. In addition, further study should be conducted to assess the hazardous effect of NPs before their widespread manufacture and usage in agricultural applications.

Although biogenic NPs are often considered to be safer than other types of nanomaterials, there are still several potential risks associated with their release into the environment. In particular, the toxic effects of metals such as Ag, Cu, and Zn on non-target organisms are still not clear to researchers [164]. To counter the toxicity issues related to NPs, the risks of this obstacle can be overcome by choosing metal ions with slight or no toxicity in low concentrations in the development of the NPs [165]. For instance, Se is found to be less toxic and more biologically active in its reduced nano-form compared with its other chemical forms, such as sodium selenite and selenium sulfide [166,167,168]. To examine the toxicity of nano-selenium, a safety evaluation demonstrated that functional selenium NPs (TSNPs) synthesized by culture filtrates of *T. harzianum* were not toxic to the human hepatic stellate cell line LX-2, the intestinal epithelial cell line HIEC, and the gastric epithelial cell line GES-1 at 50 μg/mL [50]. The Se NPs have been used in many services in the fields of industry, medicine, and agriculture in addition to their effective role as antibacterial and antifungal agents [162,169,170]. Additionally, Se NPs derived from *Trichoderma* have been demonstrated to control a broad spectrum of fungal phytopathogens. Se NPs have been synthesized by derivatives of *T. harzianum* JF309, and through triple TOF UPLC/HRMS analysis, the presence of organic compounds such as heptonic acid, ferulate, fumaric acid, and threonic acid as well as glucose and mannitol on the surface of biosynthesized SNPs was observed, which play a great role as agents that stabilize and increase the antagonistic potential of biosynthesized Se NPs significantly better than traditionally produced Se NPs against plant pathogens such as *A. alternata* XJa1, *F. verticillioide* BJ6, and *F. graminearum* PH1 [145]. Synthesized selenium NPs from cell-free culture filtrate of six species of *Trichoderma*, namely *T. asperellum*, *T. harzianum*, *T. atroviride*, *T. virens*, *T. longibrachiatum* and *T. brevicompactum*, have suppressed the growth, sporulation, and zoospore viability of *Sclerospora graminicola* under greenhouse conditions [31]. Tomato plant seeds treated with Se NPs synthesized using extracellular filtrates from *T. atroviride* (Tri_AtJSB2) showed significant protection against late blight disease caused by *P. infestans* through cellular and biochemical defense responses incited by Se NPs in tomato plants [50]. The Se NPs synthesized using the *Trichoderma* sp. cell-free culture filtrate has been exhibited to be very effective as larvicidal and antifeedant agents and can be used for the control of the *S. litura* larvae that cause damage to economic crops [51]. There is little research on the synthesis of Se NPs compared with Ag NPs. However, Se NPs synthesized by extracts of *Trichoderma* can be considered an alternative approach in the safe and economical production of NPs instead of using other salts.

## 9. Conclusions

The biosynthesis of NPs using *Trichoderma* derivatives has received a lot of interest because it is considered a green method that is simple, fast, non-toxic, and environmentally friendly. In addition, it can control the size and shape of extracellularly synthesized NPs. It was also found that the large amounts of proteins and biologically active secondary metabolites secreted naturally by these species contribute to the production of NPs in larger and safer quantities and provide clear synergistic support with the NPs in suppressing plant pathogens. On the other hand, Se is a promising candidate for NP production due to its low toxicity and high bioavailability. The integration of *Trichoderma* and their antimicrobial secondary metabolites with the low toxicity of biologically important Se could revolutionize the agricultural sector by producing biogenic Se NPs that are safe and effective for use in commercial products. Indeed, the biosynthesis of Se NPs assisted by the bioactive substrates derived from *Trichoderma* holds significant rationale to attract researchers’ attention to perform many future studies. The use of non-pathogenic *Trichoderma* spp. in the innate synthesis of Se NPs may be a futuristic reality that can be produced in large quantities for marketing purposes as nanopesticides that provide solutions in pest control and other applications that may contribute to reducing environmental pollution and to the development of biotechnology, medicine, and healthcare at reasonable costs.
